# Secondary Metabolites and the Risks of *Isaria fumosorosea* and *Isaria farinosa*

**DOI:** 10.3390/molecules24040664

**Published:** 2019-02-13

**Authors:** Qunfang Weng, Xiaofeng Zhang, Wei Chen, Qiongbo Hu

**Affiliations:** Key Laboratory of Bio-Pesticide Innovation and Application of Guangdong Province, College of Agriculture, South China Agricultural University, Guangzhou 510642, China; wengweng@scau.edu.cn (Q.W.); 13539835580@163.com (X.Z.); cwei@stu.scau.edu.cn (W.C.)

**Keywords:** *Isaria fumosorosea*, *Isaria farinosa*, secondary metabolites, risk

## Abstract

*Isaria fumosorosea* and *Isaria farinosa* are important entomopathogenic fungi with a worldwide distribution and multiple host insects. However, the concerns about the safety risks of myco-pesticides have been attracting the attention of researchers and consumers. Secondary metabolites (SMs), especially the mycotoxins, closely affect the biosafety of *Isaria* myco-insecticides. In the last forty years, more than seventy SMs were identified and isolated from *I. fumosorosea* and *I. farinose*. The SMs of *I. fumosorosea* include the mycotoxins of non-ribosomal peptides (NRPs) (beauvericin and beauverolides), terpenes (trichocaranes and fumosorinone), lactone compounds (cepharosporolides), acids (dipicolinic acid and oxalic acid), etc. Meanwhile, the NRP mycotoxins (cycloaspeptides) and the terpene compounds (farinosones and militarinones) are the main SMs in *I. farinosa*. Although several researches reported the two *Isaria* have promised biosafety, the bioactivities and the safety risks of their SMs have not been studied in detail so far. However, based on existing knowledge, most SMs (i.e., mycotoxins) do not come from *Isaria* myco-insecticide itself, but are from the host insects infected by *Isaria* fungi, because only the hosts can provide the conditions for fungal proliferation. Furthermore, the SMs from *Isaria* fungi have a very limited possibility of entering into environments because many SMs are decomposed in insect cadavers. The biosafety of *Isaria* myco-insecticides and their SMs/mycotoxins are being monitored. Of course, SMs safety risks of *Isaria* myco-insecticides need further research.

## 1. Introduction

*Isaria fumosorosea* and *Isaria farinosa*—formerly known as *Paecilomyces fumosoroseus* and *Paecilomyces farinosus*, respectively—are important entomopathogenic fungi with a worldwide distribution and multiple host insects [1,2]. Although differing from the popular *Beauveria bassiana* and *Metarhizium anisopliae* species thoroughly researched in various areas, both *I. fumosorosea* and *I. farinosa* attract more attention. They have multiple hosts, do not show harmful effects linked to the use of chemical pesticides, and are considered to be environmentally friendly [3]. Besides their application as pest biocontrol agents, there were some experiments indicating the both fungi have potential uses in the biotransformation of flavonoids glycosides, steroids, etc. [4,5,6]. 

*I. fumosorosea* is a species complex and mainly infects hemipteran and lepidopteron insects, such as aphids, leafhoppers, whiteflies, and the Asian citrus psyllid, etc. [7,8]. Other recently reported host insects besides hemipteran and lepidopteron insects include the subterranean termites, *Coptotermes curvignathus* and *Coptotermes gestroi* [9], rice weevils, *Sitophilus oryzae* [10], yellowmargined leaf beetles, and *Microtheca ochroloma* [11]. *I. fumosorosea* has been used as a pest biocontrol agent in many countries. In the USA, it was registered under NOFLY™ Technical for use as biocontrol for whiteflies, aphids, thrips, psyllids, mealybugs, and fungus gnats in greenhouses (https://www.epa.gov/pesticides/). The Apopka 97 strain in the European Union (http://ec.europa.eu/food/plant/pesticides/eu-pesticides-database/) and the Challenger or Puma myco-pesticide in Brazil (http://agrofit.agricultura.gov.br/agrofit_cons/) were registered as well. In China, although this fungus has not been registered as a myco-pesticide, it is researched and used widely to control whiteflies and aphids [3,12,13]. Compared to *I. fumosorosea*, *I. farinosa* is less researched and used. However, in the former Soviet Union, this species (*Paecilomyces farinosus*) was used as myco-insecticide named “Paecilomin” to control the apple moth, Siberian pine caterpillar, and larch caterpillar [14,15,16]. It was also reported that, in the laboratory or greenhouse, this fungus is effective against the rice weevil *Sitophilus oryzae* [10], termite *Nasutitermes corniger* [2], horn fly *Haematobia irritans* [17], two spotted spider mite *Tetranychus urticae* [18], vine mealybug *Planococcus ficus* [19], sunn pests *Eurygaster integriceps* and *Eurygaster austriaca* [20], emerald ash borer *Agrilus planipennis* [21], *Aelia rostrata* [22], pine bark-weevil (*Pissodes punctatus*) [23], etc. However, in China, *I. farinosa* is also considered as a pathogen of *Hepialus* sp. and seriously affects the production of the traditional medicinal mushroom, *Ophiocordyceps sinensis*. This is because *Hepialus* sp. is the host of *O. sinensis* [24,25,26].

The biology, ecology, and application for biocontrol agents of both fungi were carefully reviewed 10 years ago [7]. However, during the past decade, numerous research reports about the two fungal species covering various areas were published. In the area of secondary metabolites (SMs), a lot of new compounds have been isolated and identified from the fungi. Some of the SMs are mycotoxins, which have risks contaminating foods and impacting human health. There were several reports on the risk evaluations of myco-pesticides and their mycotoxins, mainly involving *B. bassiana*, *M. anisopliae*, and their NRP (non-ribosomal peptide) and PK (polyketide) metabolites [27,28,29,30]. However, there have been few documents about the risks of *Isaria* fungi and their mycotoxins. In the current review, we will focus on the mycotoxins of both *Isaria* fungi, including their structures, bioactivities, and toxicities. We will also focus on the risk evaluation of these fungi entering food chains.

## 2. Secondary Metabolites (SMs) from *Isaria fumosorosea*

**Figure 1:**
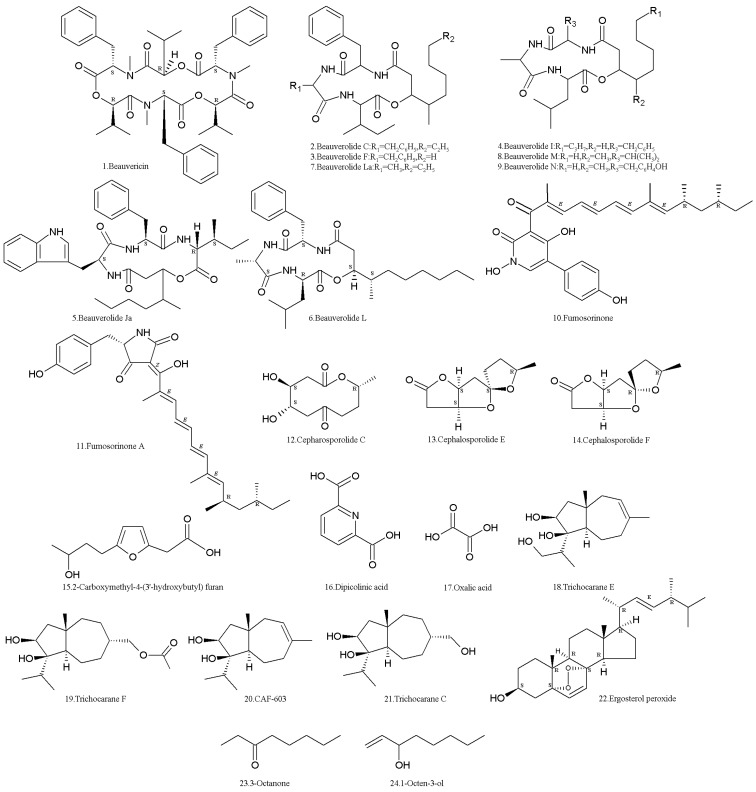
Structures of secondary metabolites (SMs) isolated from *Isaria fumosorosea*.

The NRP metabolite, beauvericin (**1**) (Table 1, Figure 1), was isolated from the strain ACCC37775 of *I. fumosorosea* (Hebei University, Baoding, China). It showed apparent inhibitory activity to protein tyrosine phosphatase 1B (PTP1B) with an IC_50_ value of 0.59 M [31]. It is a cyclic hexadepsipeptide mycotoxin with antibacterial, insecticidal, antiviral, and cytotoxic activities, and has potential value in the development of new pesticides [32,33,34,35]. However, the risks of beauvericin contamination are attracting the attention of researchers [36,37,38].

The other NRPs, beauverolides C (**2**), F (**3**), I (**4**), Ja (**5**), L (**6**), M (**8**), and N (**9**) (Table 1, Figure 1), were isolated from the strain BMFM-UNAM 834 of *I. fumosorosea* (Universidad Nacional Autonoma de Mexico, Mexico City, Mexico). These cyclotetradepsipeptides displayed a high affinity to calmodulin (CaM), with dissociation constants (Kd) ranging from 0.078–3.440 μM. Beauverolide Ja (**5**) which is the only one containing a tryptophan residue in its structure showed the highest affinity to CaM [39]. The beauverolides L and La (**7**) (Table 1, Figure 1) were isolated and identified from the PFR97-Apopka (ATCC 20874) strain (WR Grace & Co, Conn, Columbia, MD, USA) of *I. fumosorosea* [40]. Beauverolide L has anti-immunity activity against the greater wax moth, *Galleria mellonella* [41].

The terpene compound, fumosorinone (**10**) (Table 1, Figure 1) was isolated from the ACCC37775 strain of *I. fumosorosea* (Hebei University, Baoding, China). Fumosorinone (**10**) is structurally similar to tenellin and desmethylbassianin, but has different chain length and degree of methylation. Fumosorinone (**10**) acts as a classic non-competitive inhibitor of protein tyrosine phosphatase 1B (PTP1B) with an IC_50_ of 14.04 µM, which suggests that it is a potential medicine for the treatment of type II diabetes and other associated metabolic syndromes. The gene cluster of fumosorinone biosynthesis includes a hybrid polyketide synthase–nonribosomal peptide synthetase gene, two cytochrome P450 enzyme genes, a trans-enoyl reductase gene, and other two transcription regulatory genes [42]. Fumosorinone (**10**) also showed cytotoxic against human cancer lines, including HeLa, MDA-MB-231, and MDA-MB-453 cell lines [43]. A compound similar to fumosorinone, fumosorinone A (**11**) (Table 1, Figure 1), was identified as well. It is also a PTP1B inhibitor [31].

The lactone compounds, cepharosporolides C (**12**), E (**13**), and F (**14**), and an organic acid, 2-carboxymethyl-4-(3′-hydroxybutyl) furan (**15**) (Table 1, Figure 1) were isolated [31]. They had no antimalaria activity to *Plasmodium falciparum* K1 [44], and no inhibition to PTP1B [31].

The other acids, dipicolinic acid (DPA) (**16**) and oxalic acid (OXA) (**17**) (Table 1, Figure 1), were found in the *I. fumosorosea* Pfrd strain (Centro Nacional de Referencia de Control Biológico, Tecomán, Colima, Mexico). DPA (**16**) was the most abundant metabolite with insecticidal activity against the third-instar nymphs of the whitefly in bioassays involving topical applications. DPA (**16**) was detected after 24 h when the fungus started growing in submerged cultures. The production of DPA (**16**) was directly correlated with fungal growth, but the maximal yield was only 0.041 g/L [45]. In submerged fermentation, carbon was significantly directed towards the synthesis of DPA (**16**) and OXA (**17**), especially under zinc limitation [46]. OXA (**17**) has antimicrobial and antioxidant activities [47,48] and can delay the sclerotial formation of *Polyporus umbellatus* [49], which is called as “Zhuling”, a traditional Chinese medicine used for a wide range of ailments related to the edema, scanty urine, vaginal discharge, urinary dysfunction, jaundice, and diarrhea [50].

Two new carotane-type sesquiterpenes named trichocaranes E (**18**) and F (**19**), along with two known ones called CAF-603 (**20**) and trichocarane C (**21**) (Table 1, Figure 1), were isolated from the *I. fumosorosea* ACCC37775 strain (Hebei University, Baoding, China). Compounds **18**–**20** showed potent cytotoxic activities against six tumor cell lines (i.e., MDA, MCF-7, SKOV-3, Hela, A549, and HepG2), with IC_50_ values of 0.1–6.0 μg/mL [51].

Peroxy-ergosterol (**22**) (Table 1, Figure 1) was isolated from the RCEF1253 strain of *I. fumosorosea* (Anhui Agricultural University, Hefei, China) by high-speed-counter-current chromatography [52]. It has various bioactivities, such as cytotoxicity to cancer cells P-388, KB, A549, and HT-29, with ED50s of 0.4, 2.1, 2.7, and 1.4 μg/mL [53]. It also induces the apoptosis of the human leukemia cell HL-60 [54]. Furthermore, this fungus has a relatively high vitamin A content, which shows that it is a potential producer of vitamin A [55].

**Figure 2:**
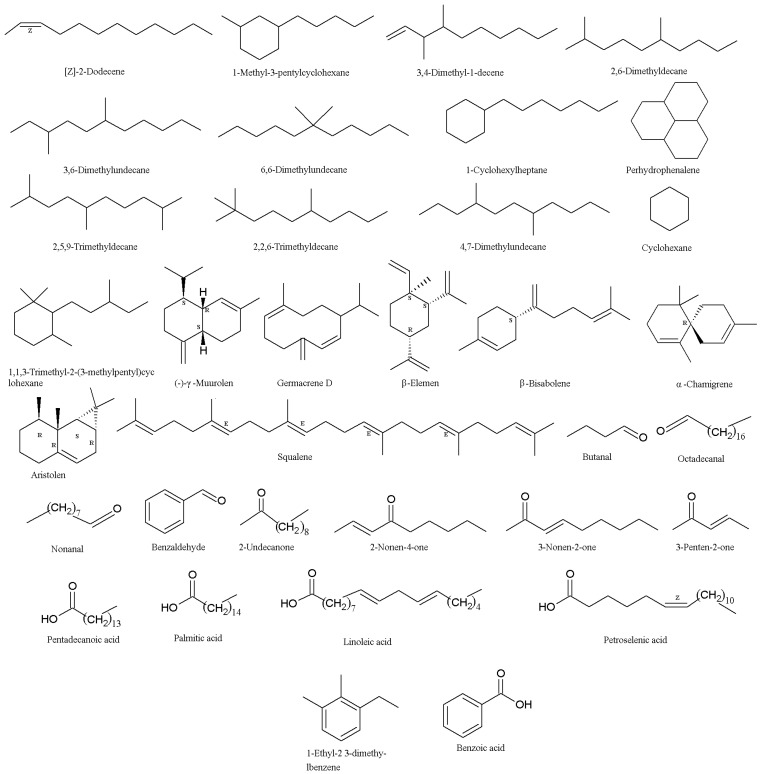
Structures of volatile organic compounds (VOCs) from *Isaria fumosorosea*.

In order to investigate the termite’s response to entomopathogenic fungi, the fungal volatile organic compounds (VOCs) were detected using GC–MS. It was found that 3-octanone (**23**) and 1-octen-3-ol (**24**) (Table 1, Figure 1) were the major surface chemical compounds on the conidia of the *I. fumosorosea* K3 strain (Kyoto University, Kyoto, Japan), and the total quantities of the two chemicals on the surface of fungal conidia were estimated to be approximately 0.01 ng per 10^7^ conidia. The Formosan subterranean termites, *Coptotermes formosanus*, showed aversion to the two compounds [56]. The results indicated that, in contrast to their reaction to *M. anisopliae*, *I. fumosorosea* cultures were not repellent to Formosan subterranean termite workers, which were highly susceptible to infection of *I. fumosorosea*. The electroantennographic responses of workers to the conidia of *I. fumosorosea* were approximately 78% less than those to *M. anisopliae*. The VOC profile of repellent cultures of *M. anisopliae* mainly consisted of paraffins (60.97%), while the major proportion of the *I. fumosorosea* profile consisted of branched and cyclic alkanes (84.41%), such as [*Z*]-2-dodecene, 1-methyl-3-pentyl cyclohexane, 3,4-dimethyl-1-decene, 2,6-dimethyldecane, 3,6-dimethylundecane, 6,6-dimethylundecane, 1-cyclohexylheptane, perhydrophenalene, 2,5,9-trimethyldecane, 2,2,6-trimethyldecane, 4,7-dimethylundecane, cyclohexane, and 1,1,3-trimethyl-2(3-methylpentyl) (Figure 2) [57]. In another report, numerous VOCs were identified in the mycelia of the *I. fumosorosea* UPH48 strain (Siedlce University of Natural Sciences and Humanities, Siedlce, Poland). The VOCs included terpenes such as γ-muurolen, germacrene D, β-elemen, β-bisabolen, α-chamigren, aristolen, and squalene. The aldehydes included butanal, octadecanal, nonanal, benzaldehyde, fenyloacetaldehyde, and 2-undecanone. The ketones included 2-nonen-4-one, 3-nonen-2-one, and 3-penten-2-one. In addition, five fatty acids (i.e., pentadecanoic, palmitic, g-linolenic, linoleic, and petroselinic) and others compounds (i.e., 1-ethyl-2,3-dimethylbenzene and benzoic acid) (Figure 2) were identified in the mycelia of the *I. fumosorosea* UPH48 strain (Siedlce University of Natural Sciences and Humanities, Siedlce, Poland) [58].

## 3. Secondary Metabolites (SMs) from Isaria farinosa

**Figure 3:**
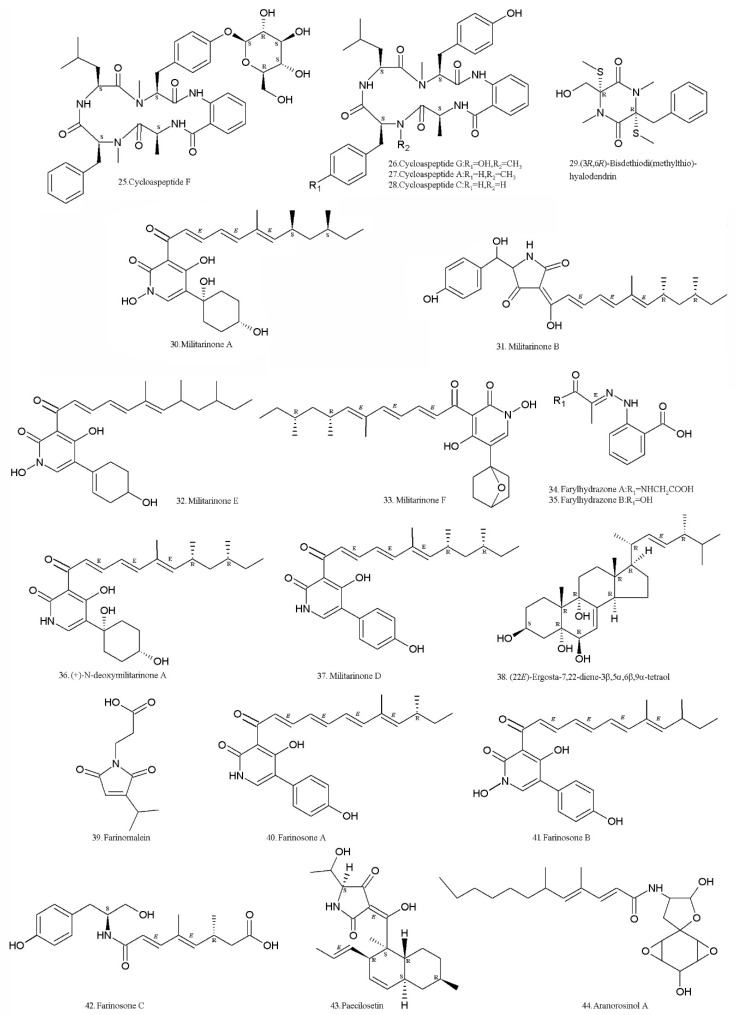
Structures of secondary metabolites (SMs) isolated from *Isaria farinosa*.

Cycloaspeptides F (**25**) and G (**26**) (Table 2, Figure 3), two new cyclic pentapeptides, and the known cycloaspeptides A (**27**), C (**28**), and bisdethiodi (methylthio) hyalodendrin (**29**) (Table 2, Figure 3) were isolated from the fermented rice substrate with the *I. farinosa* strain XJC04-CT-303 (Institute of Microbiology, Chinese Academy of Sciences, Beijing, China) that colonizes *Cordyceps sinensis*. Cycloaspeptides F (**25**) and G (**26**) inhibited the growth of MCF7 cells, which was comparable to the positive control 5-fluorouracil. They also had modest cytotoxic effects on HeLa cells [59]. Cycloaspeptide A (**27**) has a low cytotoxicity in human lung fibroblasts [60]. Cycloaspeptide C (**28**) is closely related to cycloaspeptide G (**26**), but its bioactivity is not reported. The gene cluster responsible for the biosynthesis of the cycloaspeptides were identified in *Penicillium soppii* and *Penicillium jamesonlandense*. Heterologous expression in *Aspergillus oryzae* has demonstrated that the minimal gene set required to produce both cycloaspeptide A and cycloaspeptide E is a 5-module NRPS and a new type of pathway-specific *N*-methyltransferase (N-MeT). Gene knock-outs and feeding studies have demonstrated that two modules of the NRPS preferentially accept and incorporate *N*-methylated amino acids, which are provided by the pathway-specific N-MeT. This is a system not previously seen in secondary metabolism [61]. The diketopiperazine derivative, bisdethiodi (methylthio) hyalodendrin (**29**) (gliovictin), was isolated in 1973. It exhibited weak cytotoxic activity on KB (human epidermoid carcinoma of the mouth) with IC_50_ of 42 µg/mL, and had IC_50_ values of >50 µg/mL on HepG2 (human hepatocellular liver carcinoma cell line), A549 (human lung carcinoma cell line), HCC-S102 (hepatocellular carcinoma cell line), HuCCA-1 (human cholangiocarcinoma cancer cells), HeLa (cervical adenocarcinoma cell line), MDA-MB231 (human breast cell line), T47 D (human mammary adenocarcinoma cell line), HL-60 (human promyelocytic leukemia cell line), and P388 (murine leukemia cell line) [62].

The terpene compounds, militarinones A (**30**), B (**31**), E (**32**), and F (**33**) (Table 2, Figure 3), were isolated from the *I. farinosa* strain XJC04-CT-303 (Institute of Microbiology, Chinese Academy of Sciences, Beijing, China) [63]. Militarinones A (**30**) and E (**32**) had significant cytotoxicity on the A549 human carcinoma cell line, whereas militarinone B (**31**) was active against *Staphylococcus aureus*, *Streptococcus pneumoniae*, and *Candida albicans* [63]. Militarinone A (**30**) at the concentration of 10 μM, had obvious neurotrophic effects on PC-12 cells [64]. The phenylhydrazones, farylhydrazones A (**34**) and B (**35**) (Table 2, Figure 3), were isolated from this strain [63]. No bioactivity report has been found yet. The two compounds can be synthesized with six and five steps respectively, starting from 2-nitrobenzoic acid [65].

A new pyridone alkaloid, (+)-*N*-deoxymilitarinone A (**36**), along with the related metabolites, militarinone D (**37**), militarinone B (**31**) and the sterol 22*E*,4*R*-ergosta-7,22-diene-3β,5α,6β,9α-tetraol (**38**) (Table 2, Figure 3), were isolated from *I. farinosa* RCEF 0097 (Entomogenous Research Centre, Anhui Agricultural University, Hefei, China). The (+)-*N*-deoxymilitarinone A (**36**) at 33 and 100 μM concentrations induced neurite sprouted in PC 12 cells. A cytotoxic effect was observed in human neurons (IMR-32) at a concentration of 100 μM [66]. A diverted total synthesis approach to the total synthesis of *N*-deoxymilitarinone A was developed by Ding et al. [67].

A new maleimide-bearing compound, farinomalein (**39**) (Table 2, Figure 3), was isolated from the strain HF599 (National Institute of Fruit Tree Science, Tsukuba, Japan). It showed potent activity against the plant pathogen *Phytophthora sojae* [68]. It can be synthesized in two steps from a readily available γ-hydroxybutenolide [69]. The activities against *Phytophthora sojae* and *Aphanomyces cochlioides* were confirmed [70]. 

Two new yellow pigments, farinosones A (**40**) and B (**41**), together with farinosone C (**42**) (Table 2, Figure 3), were isolated from the mycelial extract of the entomogenous fungal strain RCEF 0101 (Entomogenous Research Centre, Anhui Agricultural University, Hefei, China). Farinosone C (**42**) is a new metabolite derived from an early step of pyridone alkaloid biosynthesis. Farinosones A (**40**) and C (**42**) at 50 µM induced neurite outgrowth in the PC-12 cell line, while farinosone B (**41**) was inactive. The three farinosones had no cytotoxicity against PC-12 cells when tested at 50 µM concentration in the MTT assay [71]. Farinosones are similar to militarinones in structure, belonging to the terpene group of compounds.

A new tetramic acid derivative, paecilosetin (**43**) (Table 2, Figure 3), was isolated from the strain CANU TE108 (University of Canterbury, Christchurch, New Zealand). It showed activity against the P388 cell line, with IC_50_ values of 3.1 μg/mL, and was also active against the microorganisms *Bacillus subtilis*, *Trichophyton mentagrophytes*, and *Cladosporium resinae* [72]. Paecilosetin (**43**) and aranorosinol A (**44**) (Table 2, Figure 3) were identified in the *I. farinosa* HF511 strain. They act plant pathogenic oomycetes, *Phytophthora sojae* and *Aphanomyces cochlioides* [70]. Aranorosinol A (**44**) also had weak antibacterial and antifungal properties on other microorganisms [73].

*I. farinosa* can produce a water soluble anthraquinone-related red pigment with good stability after being exposed to salt solution (96.1% stability after treatment with sodium chloride), acid (72.1% stability with citric acid), heat (86.2% stability at 60 °C), and sunlight (99.4% stability). It shows a potential for pigment production [74,75]. 

## 4. Risks of the Secondary Metabolites (SMs) from both *Isaria* Myco-Insecticides

In recent years, the concerns about the safety risks of myco-pesticides and their SMs have been attracting the attention of researchers and consumers. In fact, the popular myco-insecticides, *Beauveria bassiana*. and *Metarhizium anisopliae*, have been proposed as low-risk environmental alternatives to chemical insecticides for controlling agricultural pests and disease vectors [27,28] This is because their safety for humans and the environment were well evaluated [76,77,78,79], while the mycotoxins they produced were considered unlikely to enter food chains [80]. 

However, there were few experiments on the safety of *Isaria* myco-insecticides. *I. fumosorosea* was the subject of two reports involving safety analysis, while *I. farinosa* has not been paid attention yet. The *I. fumosorosea* monospore culture EH-506/3 (BMFM-UNAM 834, Universidad Nacional Autonoma de Mexico, Mexico City, Mexico) was subjected to a biosafety test by applying a 2 g/kg of animal body weight dose on the shaved skin of 16 New Zealand rabbits, with an exposure time of 24 h. The results indicated that none of the rabbits showed clinical signs of any disease, and their body weight corresponded to the expected weight for a healthy rabbit. The test data supports the safety of *I. fumosorosea* EH-506/3 when applied to the skin [29]. Another toxicity test on *I. fumosorosea* was completed in China. The toxicities of acute oral, dermal, and inhalation to rats were recorded as LD_50_ > 5000 mg/kg, LD_50_ (4 h) > 2000 mg/kg, and LC_50_ (2 h) > 2000 mg/m^3^, respectively. No irritation action was observed in rabbit eyes, and no dermal sensitization reaction was found on the treated rabbit skin. These results suggested that *I. fumosorosea* has low toxicities of acute oral, dermal toxicity, and inhalation, and it can be graded as a weak sensitizer [81].

**Figure 4:**
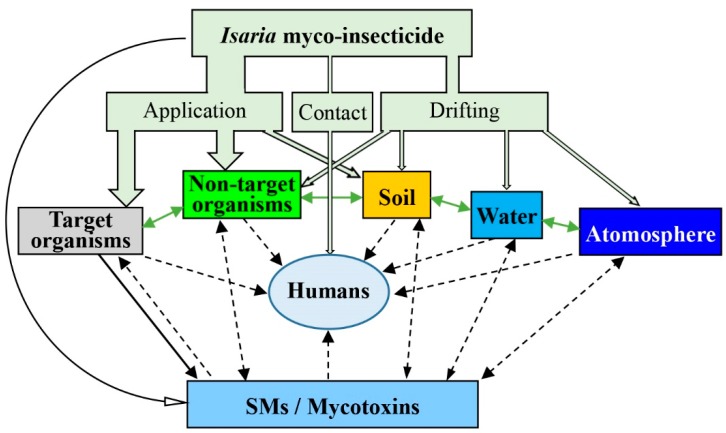
The sources and destinations of the *Isaria* myco-insecticide and its mycotoxins. 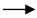 indicates an existing pathway, while 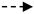 indicates a pathway that has not been found to date. (SMs = secondary metabolites) (modified based on Hu et al. (2016) [81]).

Overall, there are six destinations (i.e., target organisms, non-target organisms, soil, water, atmosphere, and humans) involved in the production and application of *Isaria* myco-insecticide formulations (Figure 4). The most important destination is target organisms, including the pests and crops, when *Isaria* myco-insecticide is released in fields. In practice, a few fungal spores of myco-insecticide probably land on insect surfaces, but the fungus will proliferate on the infected insect hosts, which suffer a pathogenic process from spore germination and the formation of the next generation of spores. Many mycotoxins of entomopathogenic fungi are probably biosynthesized in hemocoel to conquer the host’s immunity [82]. The target crops are probably the main destination, especially when the *Isaria* myco-insecticide is applied by stem-leaf treatment in fields. This is because most of the fungal spores are dropped on the plants with canopy covering the ground. In addition, the endophytic characteristics of entomopathogenic fungi might produce some SMs because *Isaria* fungi, similar to *B. bassiana* and *M. anisopliae*, can colonize plants [83,84,85]. Non-target organisms are an important destination of myco-insecticides as well. They represent a big category, including animals, plants, and microbes, which are not the targets of myco-insecticides but have chances to contact myco-insecticides. Among them, non-target insects might be the most important destination, because some of these insects probably are the hosts of myco-insecticidal fungi. There have been many reports published giving evidence that entomopathogenic fungi infect silkworms [86], bees [87,88], and natural enemy insects [89]. Of course, more studies found that the myco-insecticides are safe to non-target insects if they are used correctly [90,91].

Soil is another important destination, especially when *Isaria* myco-insecticide is released through soil treatments in fields (Figure 4). Through the drifting pathway from application and dropping pathway from target pest cadavers, fungal phages and mycotoxins can enter the soil system. In fact, entomopathogenic fungi can persist and survive in soil for a long time [92,93,94,95]. *Beauveria* spp., *Metarhizium* spp., *Paecilomyces* spp., and *Isaria* spp. can be often isolated from the soil [1]. The entomopathogens in soil can be detected after myco-insecticides are used [96], but there are no reports about the growth and proliferation of *Isaria* fungi and the presence of their SMs in soil. There are also no cases of soil fungi affecting human health.

Water and atmosphere are the destinations of the drifting myco-insecticides (Figure 4). In fact, many entomopathogenic fungi can persist and survive in water. *I. farinosa* and *I. fumosorosea*, similarly to *M. anisopliae*, can infect aquatic insects like mosquitoes [97,98]. However, there are few studies about SMs of entomopathogenic fungi in water. Milner et al. reported that the *Metarhizium* biopesticide is very unlikely to pose any hazard to aquatic organisms [99]. The atmosphere is another destination of *Isaria* myco-insecticides, where fungal entomopathogens are obtained from drifting myco-insecticides and the spore dispersal of natural fungi. However, fungi cannot either persist for a long time or proliferate in the air [100]. Also, fungi might be exchanged between soil, water, and atmosphere systems, although there are few studies on this aspect. To the date, there are also no cases of entomopathogenic fungi from water and atmosphere influence on human health.

Humans contact *Isaria* myco-insecticides through direct and indirect ways (Figure 4). Only people who are involved in the production process of myco-insecticides or use them in farms directly contact the fungi. Several studies have reported that fungal spores lead to allergies in workers who worked in factories of *B. bassiana* and *M. anisopliae* for long periods [27,28]. Perhaps, most people indirectly contact the fungi through foods, soil, the atmosphere, and water contaminated by the fungi. However, there have not been any case reports about people’s health affected by indirectly contacting entomopathogenic fungi.

Undoubtedly, the biosafety risks of *Isaria* myco-insecticides are closely related to the sources and fates of the SMs (especially the mycotoxins) produced by entomopathogenic fungi.

In fact, the SMs of myco-insecticide itself are very limited. Because the active ingredients of myco-insecticide formulations are the spores of the fungal entomopathogen, the fungi cannot proliferate in the formulation and cannot produce new SMs. Most SMs possibly exist in the spore cells rather than outside the spores [80]. Therefore, the main sources of SMs basically include the target pests or host insects infected by fungal entomopathogen of myco-insecticide, because the host insects support the fungi with conditions for proliferation. The source of SMs and mycotoxins is the growing entomopathogenic filamentous fungus. In addition, endophytic entomopathogenic fungi might be an SM source, because some *Isaria* strains can colonize plants and become endophytic fungi [101,102]. 

Currently, we do not know the detailed fates of the fungal SMs of myco-insecticides. Obviously, there are certain possibilities that the SMs of entomopathogenic fungi enter environments, however, there have not been any reports that show evidence of the entry of SMs from myco-insecticides into environments. In fact, a few research cases indicate that mycotoxins are scarcely released into environments from insects. For example, destruxin analogues were shortly decomposed by *M. anisopliae* after the host insects died, which was presumably due to the activity of hydrolytic enzymes in the insects’ cadavers. This appeared to be independent of host or soil type and biota. The study supported that destruxins are essentially restricted to the host and pathogen and are unlikely to contaminate the environment or enter the food chain [103,104]. 

To date, it has been found that most mycotoxins that contaminate environments and food chains come from the crops and products infected by fungal phytopathogens, such as *Fusarium* spp., *Aspergillus* spp., etc., rather than fungal entomopathogens [38,105], despite the fact that both phytopathogenic and entomopathogenic fungi often produce the same mycotoxins [106]. For example, Schenzel et al. reported that beauvericins were detected in drainage water where wheat was inoculated with *Fusarium* spp., which is a producer of beauvericins [107].

In conclusion, there are more than seventy SMs identified and isolated from *I. fumosorosea* and *I. farinosa*. Many of these are mycotoxins attracting people’s concerns about the biosafety. The SMs of *I. fumosorosea* include the NRP mycotoxins (beauvericin and several beauverolides), terpenes (several trichocaranes and fumosorinone), lactone compounds (several cepharosporolides), and acids (dipicolinic acid and oxalic acid). In *I. farinosa*, the NRPs (several cycloaspeptides) and terpenes (several farinosones and militarinones) were the main SMs. Currently, the bioactivities and mechanisms of action of the SMs in both *Isaria* have not been well studied, and neither have the risks of these compounds been carefully assessed. However, it is indicated that most SMs (mycotoxins) come from the host insects infected by *Isaria* fungi rather than the *Isaria* myco-insecticide itself, because the hosts provide all the conditions for fungal proliferation. Furthermore, the possibility of SMs from *Isaria* fungi entering into environments is very limited, because many SMs are decomposed in insect cadavers. Although more careful research in the future is essential, the biosafety of *Isaria* myco-insecticides and their SMs/mycotoxins in current is under control. 

## Figures and Tables

**Table 1:** SMs from *Isaria fumosorosea* and their biological activities.

Metabolite	CAS no.	Strain	Biological activity	References
Beauvericin (**1**)	26048-05-5	ACCC37775 (Hebei University, Baoding, China)	Inhibiting HepG2 cells with an IC_50_ of 2.40 μM. Cytotoxicity to multiple drug-resistant HepG2 cell lines with an IC_50_ value 25-fold more than that of doxorubicin. Inhibitor of PTP1B with an IC_50_ of 0.59 μM.	[32,33,34,35,36]
Beauverolide C (**2**)	75899-64-8	BMFM-UNAM 834 (Universidad Nacional Autonoma de Mexico, Mexico City, Mexico).	Calmodulin (CaM) inhibitor	[40]
Beauverolide F (**3**)	75947-00-1	Same as above	Calmodulin (CaM) inhibitor	[40]
Beauverolide I (**4**)	62995-91-9	Same as above	Calmodulin (CaM) inhibitor	[40]
Beauverolide Ja (**5**)	76265-41-3	Same as above	Calmodulin (CaM) inhibitor	[40]
Beauverolide L (**6**)	154491-56-2	BMFM-UNAM 834 (Universidad Nacional Autonoma de Mexico, Mexico City, Mexico); PFR97-Apopka (ATCC 20874) (WR Grace & Co, Conn, MD, USA)	Calmodulin (CaM) inhibitor Anti-immunity activity against the greater wax moth, *Galleria mellonella*	[40,41,42]
Beauverolide La (**7**)	160825-68-3	PFR97-Apopka (ATCC 20874) (WR Grace & Co, Conn, MD, USA)		[41]
Beauverolide M (**8**)		BMFM-UNAM 834 (Universidad Nacional Autonoma de Mexico, Mexico City, Mexico).	Calmodulin (CaM) inhibitor	[40]
Beauverolide N (**9**)		Same as above	Calmodulin (CaM) inhibitor	[40]
Fumosorinone (**10**)	1879030-70-2	ACCC37775 (Hebei University, Baoding, China)	Inhibitor of PTP1B (IC_50_ of 14.04 μM)	[43,44]
Fumosorinone A (**11**)	2241028-99-7	Same as above	Inhibitor of PTP1B (IC_50_ of 3.24 μM)	[32]
Cepharosporolide C (**12**)	97344-02-0	Same as above	No activities to malaria *Plasmodium falciparum* K1, and PTP1B	[32,45]
Cepharosporolide E (**13**)	97373-15-4	Same as above		[32,45]
Cepharosporolide F (**14**)	97344-04-2	Same as above		[32,45]
2-carboxymethyl-4-(3′-hydroxybutyl)furan (**15**),		Same as above		[32,45]
Dipicolinic acid (**16**)	499-83-2	Pfrd (Centro Nacional de Referencia de Control Biológico, Tecomán, Colima, Mexico)	Insecticidal activity against third-instar whitefly nymphs	[47,48,49,50]
Oxalic acid (OXA) (**17**)	144-62-7	Same as above	Insecticidal activity against third-instar whitefly nymphs	[47,48,49,50]
Trichocarane E (**18**)		ACCC37775 (Hebei University, Baoding, China)	Cytotoxicity to six tumor cell lines (i.e., MDA, MCF-7, SKOV-3, Hela, A549, and HepG2) with an IC_50_ of 0.1–6.0 μg/mL.	[52]
Trichocarane F (**19**)		Same as above	Cytotoxicity to six tumor cell lines (i.e., MDA, MCF-7, SKOV-3, Hela, A549, and HepG2, with an IC_50_ of 0.1–6.0 μg/mL.	[52]
CAF-603 (**20**)		Same as above	Cytotoxicity to six tumor cell lines (i.e., MDA, MCF-7, SKOV-3, Hela, A549, and HepG2, with an IC_50_ of 0.1–6.0 μg/mL.	[52]
Trichocarane C (**21**)		Same as above		[52]
Ergosterol peroxide (**22**)	2061-64-5	RCEF1253 (Anhui Agricultural University, Hefei, China)	Cytotoxic to cancer cells P-388, KB, A549, and HT-29 (with ED_50_ values of 0.4, 2.1, 2.7, and 1.4 μg/mL) and human leukemia cell, HL-60	[53,54,55,56]
3-octanone (**23**)	106-68-3	Conidia of strain K3 (Kyoto University, Kyoto, Japan).	Repellent to termites	[57]
1-octen-3-ol (**24**)	3391-86-4	Same as above	Repellent to termites	[57]

**Table 2:** Secondary metabolites (SMs) from *Isaria farinosa* and their biological activities.

Metabolite	CAS no.	Strain	Biological Activity	References
Cycloaspeptide F (**25**)	1174132-23-0	XJC04-CT-303 (Institute of Microbiology, Chinese Academy of Sciences, Beijing, China)	Cytotoxic to HeLa and MCF7 cell lines	[60]
Cycloaspeptide G (**26**)	1174132-24-1	Same as above	Cytotoxic to HeLa and MCF7 cell lines	[60]
Cycloaspeptide A (**27**)	109171-13-3	Same as above	Cytotoxicity to human lung fibroblasts	[60,61]
Cycloaspeptide C (**28**)	109171-15-5	Same as above		[60]
(3*R*,6*R*)-Bisdethiodi (methylthio) hyalodendrin (**29**)	52080-06-5	Same as above	Weak cytotoxic activity	[60,63]
Militarinone A (**30**)	400604-05-9	Same as above	Cytotoxicity to A549 cells. Neurotrophic effects on PC-12 cells	[64,65]
Militarinone B (**31**)	503584-83-6	RCEF0097 (Anhui Agricultural University, Hefei, China); XJC04-CT-303 (Institute of Microbiology, Chinese Academy of Sciences, Beijing, China)	Anti-microbes to *Staphylococcus aureus*, *Streptococcus pneumoniae*, and *Candida albicans*	[64]
Militarinone E (**32**)	1261060-55-2	XJC04-CT-303 (Institute of Microbiology, Chinese Academy of Sciences, Beijing, China)	Cytotoxicity to A549 cells	[64]
Militarinone F (**33**)	1261060-56-3	Same as above		[64]
Farylhydrazone A (**34**)	1261060-57-4	Same as above		[66]
Farylhydrazone B (**35**)	1261060-58-5	Same as above		[66]
(+)-*N*-deoxymilitarinone A (**36**)	881376-40-5	RCEF0097 (Anhui Agricultural University, Hefei, China)	Induce neurite sprouting in PC 12 cells when tested at 33 and 100 μM concentrations. Cytotoxic to human neurons (IMR-32) at a concentration of 100 μM.	[67]
Militarinone D (**37**)	503584-82-5	RCEF 0097 (Anhui Agricultural University, Hefei, China); XJC04-CT-303 (Institute of Microbiology, Chinese Academy of Sciences, Beijing, China)		[67]
(22*E*)-Ergosta-7,22-diene-3β,5α,6β,9α-tetraol (**38**)	88191-06-4	Same as above		[67]
Farinomalein (**39**)	1175521-35-3	HF599 (National Institute of Fruit Tree Science, Tsukuba, Japan)	Antifungal to phytopathogenic *Phytophthora sojae*	[69,70,71]
Farinosone A (**40**)	816431-89-7	RCEF 0101 (Anhui Agricultural University, Hefei, China)	Neuritogenic in the PC-12 cell model	[72]
Farinosone B (**41**)	816431-94-4	Same as above	Inhibitory to *Bacillus subtilis* and *Staphylococcus aureus*. Moderate cytotoxicity to brine shrimp larvae (*Artemia salina*)	[72]
Farinosone C (**42**)	816431-98-8	Same as above	Induced neurite outgrowth in the PC-12 cell line at concentrations of 50 μM	[72]
Paecilosetin (**43**)	856258-89-4	CANU TE108 (University of Canterbury, Christchurch, New Zealand). HF511 (National Institute of Fruit Tree Science, Tsukuba, Japan.)	Antioomycete activity against both *Phytophthora sojae* and *Aphanomyces cochlioides*	[71,73]
Aranorosinol A (**44**)	145147-04-2	HF511 (National Institute of Fruit Tree Science, Tsukuba, Japan.)	Antioomycete to both *Phytophthora sojae* and *Aphanomyces cochlioides*	[71,74]
